# Cranial fontanelles and the association of bregmatic fontanelle width with cranial morphology in newborn Chihuahua and non-brachycephalic puppies

**DOI:** 10.3389/fvets.2026.1922844

**Published:** 2026-10-20

**Authors:** Elina Rautala, Sanna Malkamäki, Tarja S. Jokinen, Junnu Leikola, Mirja Ruohoniemi, Jouni J. T. Junnila, Anna-Mariam Kiviranta

**Affiliations:** 1 Department of Equine and Small Animal Medicine, Faculty of Veterinary Medicine, University of Helsinki, Helsinki, Finland; 2 Pathology Unit, Department of Veterinary Biosciences, Faculty of Veterinary Medicine, University of Helsinki, Helsinki, Finland; 3 Cleft Palate and Craniofacial Center, Department of Plastic Surgery, University of Helsinki and Helsinki University Hospital, Helsinki, Finland; 4 EstiMates Oy, Espoo, Finland

**Keywords:** brachycephaly, Chihuahua, craniometric measurement, fontanelle, micro-CT

## Abstract

**Introduction:**

In adult Chihuahuas, persistent fontanelles are common and have been associated with extreme brachycephaly. It is currently unknown whether persistent fontanelles are congenital bone defects or develop during cranial growth due to bone resorption.

**Objectives:**

We investigated the occurrence of cranial fontanelles, cranial shape, and relative bregmatic fontanelle width in newborn Chihuahuas and non-brachycephalic puppies.

**Methods:**

The study included 50 newborn puppy cadavers. Micro-computed tomography (CT) images of the cranium were used to evaluate occurrence of cranial fontanelles and to measure relative width of the bregmatic fontanelle. Histological samples of the cranium were used to confirm the presence of cranial fontanelles. CT images of the cranium were used to evaluate craniometric ratios, including two different cranial height to cranial base length ratios, cranial height to length, and cranial height to width ratios, and cranial index (CrI). The association of the relative width of the bregmatic fontanelle with craniometric ratios was examined.

**Results:**

19/19 Chihuahuas and 6/21 non-brachycephalic puppies had cranial fontanelles. All but one puppy had only one fontanelle, which was located at the bregma. The proportion of puppies having at least one cranial fontanelle was higher in Chihuahuas than in non-brachycephalic puppies (*p* < 0.001). Cranial height to cranial base length ratios, cranial height to length ratio, and CrI were higher in Chihuahuas than in non-brachycephalic puppies (*p* < 0.001 for each analysis). Bregmatic fontanelle was relatively wider in Chihuahuas than in non-brachycephalic puppies (*p* = 0.0013) and was associated with higher cranial height to cranial base length 2 (*p* = 0.022), and cranial height to length ratios (*p* = 0.023).

**Conclusion:**

Bregmatic fontanelle occurred more frequently in Chihuahuas than in non-brachycephalic puppies, but other cranial fontanelles were uncommon. Our findings suggest that newborn Chihuahuas differ from adult Chihuahuas in which persistent fontanelles are frequently detected. This might indicate that persistent fontanelles located outside bregma may develop during postnatal growth, with bone remodeling and resorption being one possible mechanism. Chihuahuas had brachycephalic cranium already at the time of birth. In newborn puppies, relatively wider bregmatic fontanelle was associated with a shorter and higher cranium, i.e., brachycephalic cranial shape.

## 1 Introduction

Chihuahua is a toy-sized brachycephalic dog breed. Persistent fontanelles emerge commonly in adult Chihuahuas on all surfaces of the cranium. They have been associated with low body weight, ventriculomegaly, syringomyelia, craniocervical junction overcrowding, and clinical signs related to Chiari-like malformation and syringomyelia, indicating their clinical relevance (1, 2). In adult dogs, the calvarium is expected to be fully ossified, and no persistent fontanelles should be observed (3). According to the literature, bregmatic fontanelle is the only fontanelle observed in puppies, and it closes within 1 month of age (3, 4). It is currently unknown whether cranial fontanelles other than bregmatic fontanelle occur already in newborn Chihuahuas.

Cranial fontanelles are membrane-covered areas of fibrous tissue located in the intersection of cranial sutures. Cranial fontanelles allow compression of the cranium during passage through the birth canal and enable expansion of the cranial vault as the brain grows (5, 6). During maturation, the brain growth decelerates, and the fontanelles close because of approaching bone fronts (6). Mature bony cranium protects the underlying brain tissue. Dogs differ from children who are born with six open fontanelles: two in the midline (anterior and posterior fontanelle) and two on each side of the cranium (paired sphenoid and mastoid fontanelles) (5). In adult Chihuahuas, two-thirds of persistent fontanelles are in similar locations to children, with bregmatic fontanelle being the most common persistent fontanelle (1). Currently, it is unknown whether persistent fontanelles in adult Chihuahuas represent congenital ossification defects or are caused by bone resorption during cranial growth. In dogs, cranial fontanelles have been examined by using diagnostic imaging methods, including magnetic resonance imaging (MRI), computed tomography (CT) imaging, and ultrasonography (1, 4, 7). In human fetuses, anterior fontanelle has also been examined with histological samples of the cranium (8).

Dogs are divided into brachycephalic, mesaticephalic, and dolichocephalic breeds based on their skull shape (3, 9, 10). Different indices are used to describe the width to length ratio of the skull. Skull index describes the width to length ratio of the whole head, and cranial index (CrI) width to length ratio of the neurocranium, with higher values indicating a greater degree of brachycephaly (3). Cranial growth occurs at cranial sutures, which are fibrous connections between calvarial bones. Cranial growth is directed by the growing brain and is regulated by signals from the meninges and the osteogenic fronts of the calvarial bones (11, 12). Growth of the cranial base occurs in the cartilaginous junctions (synchondroses) between cranial base bones, enabling longitudinal expansion of the cranium (3). In brachycephaly, longitudinal growth of the cranium is restricted, leading to compensatory cranial growth in width and height (3, 13, 14).

In adult Chihuahuas, extreme brachycephaly is associated with larger and more numerous persistent fontanelles. Larger total area and higher number of persistent fontanelles are associated with higher CrI and higher cranial height to cranial base length ratio, reflecting increased cranial doming (15). The Chihuahua breed standard emphasizes round cranial shape (16). However, it is unknown whether the cranial shape differs between Chihuahuas and non-brachycephalic puppies already at the time of birth. Furthermore, it is unknown whether the relative width of the bregmatic fontanelle is associated with cranial morphology in newborn puppies.

The primary aim of this study was to determine the occurrence of cranial fontanelles in newborn Chihuahuas and non-brachycephalic puppies. Our hypothesis was that newborn Chihuahuas have other cranial fontanelles besides the bregmatic fontanelle, and the number of cranial fontanelles in Chihuahuas is higher than in non-brachycephalic puppies. Our secondary aim was to evaluate differences in cranial morphology based on several craniometric ratios between newborn Chihuahuas and non-brachycephalic puppies. We hypothesized that CrI and cranial height to cranial base length ratios are higher in newborn Chihuahuas than in non-brachycephalic puppies. A third aim was to compare the relative width of the bregmatic fontanelle between newborn Chihuahuas and non-brachycephalic puppies and to evaluate the association of the relative width of the bregmatic fontanelle with cranial morphology. Our hypotheses were that the relative width of the bregmatic fontanelle is larger in Chihuahuas than in non-brachycephalic puppies and in puppies with higher CrI and cranial height to cranial base length ratios.

## 2 Materials and methods

This was a prospective cross-sectional study examining cranial fontanelles and cranial morphology in newborn Chihuahua and non-brachycephalic puppy cadavers using MRI, CT, and micro-CT imaging and histological samples of the cranium.

### 2.1 Animals

This study included full-term newborn Chihuahuas and non-brachycephalic puppies that had either died during parturition or died spontaneously or been euthanized within 6 days of parturition. Because the size of the bregmatic fontanelle diminishes with advancing age (3, 4, 17), we included puppies with maximum age of 6 days. The study included puppies either born by natural parturition or Cesarean section. The breed and age of the puppy and days of gestation were recorded. The sex of the puppy was recorded if available. Preterm puppies (duration of gestation less than 57 days) (18), brachycephalic puppies except for Chihuahuas, and puppies with macroscopically observable malformations in any parts of the body were excluded from the study. Furthermore, if MRI, CT, or necropsy revealed cranial malformations or space occupying lesions of the head, or the skull was fragile due to autolytic changes, the puppy was excluded from the study.

The puppies were obtained from Finnish dog breeders, private clinics in the capital area of Finland and the Veterinary Teaching Hospital of the University of Helsinki, Finland, between the years 2020 and 2024. An ethics statement concerning the study was issued by the local Viikki Campus Research Ethics Committee, University of Helsinki (Statement 18/2021). Participation was voluntary for the owners, and they signed a consent form when participating in the study. None of the puppies were euthanized because of the study.

Sample size calculation was based on the main aim of the study which was to evaluate if cranial fontanelles occur in newborn Chihuahuas and if Chihuahuas differ from non-brachycephalic puppies. Bregmatic fontanelle can normally be present in newborn puppies, hence, the occurrence of cranial fontanelles located outside bregma were used for sample size calculation. As information regarding newborn puppies is lacking, the percentage used in sample size calculation was an estimation. The sample size calculation was based on following assumptions: Assuming that 40 and 5% of the subjects in two groups have the factor of interest (cranial fontanelle located outside bregma), and after applying continuity correction, the study would require a sample size of 24 for each group to achieve a power of 80 for detecting difference in proportions of −0.35 between the two groups at the *p*-value of 0.05.

### 2.2 Diagnostic imaging

MRI examinations of the head were conducted in ventral recumbency with a 1.5 Tesla scanner (Philips, Ingenia 1.5 T S, Philips Medical System) using a small extremity coil. For all but one puppy undergoing MRI, turbo-Spin-Echo T2-weighted transverse (TR 3000, TE 90, slice thickness 2 mm), and sagittal (TR 2619, TE 100, slice thickness 2 mm) plane MRI was performed. In one puppy, only T2 transverse plane MRI was performed.

CT images of the head were acquired using a 64-slice helical scanner (GE LightSpeed VCT 64, GE Healthcare). The parameters used were tube voltage 120 kV, maximum current 320 mAs, interval 0.312 mm, and slice thickness 0.625 mm. In two puppies, CT images were acquired using 100 kV and 420 mAs. Images were reconstructed with bone and soft tissue algorithms.

Micro-CT imaging of the head was conducted using a Phoenix Nanotom|s device (Waygate Technologies). The X-ray tube voltage was set at 80 kV, the current was 120 μA, and a 0.1 mm Cu filter was used. For each scan, a total of 1,600 projections were recorded over a 360° rotation with 1.5 s exposure time for each projection. The voxel size was chosen based on sample size and varied between 40 μm and 57.5 μm. The reconstruction was performed using Phoenix Datos|x 2 reconstruction software (version 2.4.0). The resulting 16-bit image volumes were then converted to DICOM (Digital Imaging and Communications in Medicine) stacks using Dragonfly software (version 2022.2.0.1367).

### 2.3 Image analysis

All MRI, CT, and micro-CT images were viewed in DICOM format with an OsiriX DICOM viewer (Pixmeo Sarl, version 14.1.2, Bernex, Switzerland). The first assessor (ER) is an experienced veterinarian in radiology, and the second assessor (A-MK) is a board-certified neurologist.

### 2.4 Computed tomography and magnetic resonance imaging analysis

In all puppies, CT images of the head were evaluated for space occupying lesion in the brain which was an exclusion criterion in this study. When available, MRI images of the brain were used to complement this evaluation. MRI images were subjectively evaluated for space-occupying fluid-filled lesions such hydrocephalus or cystic lesions. Due to limited set of sequences, the MRI evaluation was limited to large lesions that could affect cranial ossification.

Computed tomography images were used to conduct several craniometric measurements to evaluate cranial morphology. Analysis was performed by one assessor (ER) from anonymized CT images with windowing allowing visualization of bone. During the analysis the assessor was blinded to the history, breed, and sex of the puppy and to the results of other imaging modalities and histological examination of the fontanelles. Craniometric measurements were conducted from CT images, as previously described (15), with some modifications described under each measurement (Figures 1A,B). Common anatomic landmarks were used for cranial length and width measurements (3, 15). Because in a previous study the measurement methods had high intra- and inter-rater reliabilities (15), in this study one evaluator performed the measurements.

**Figure 1:**
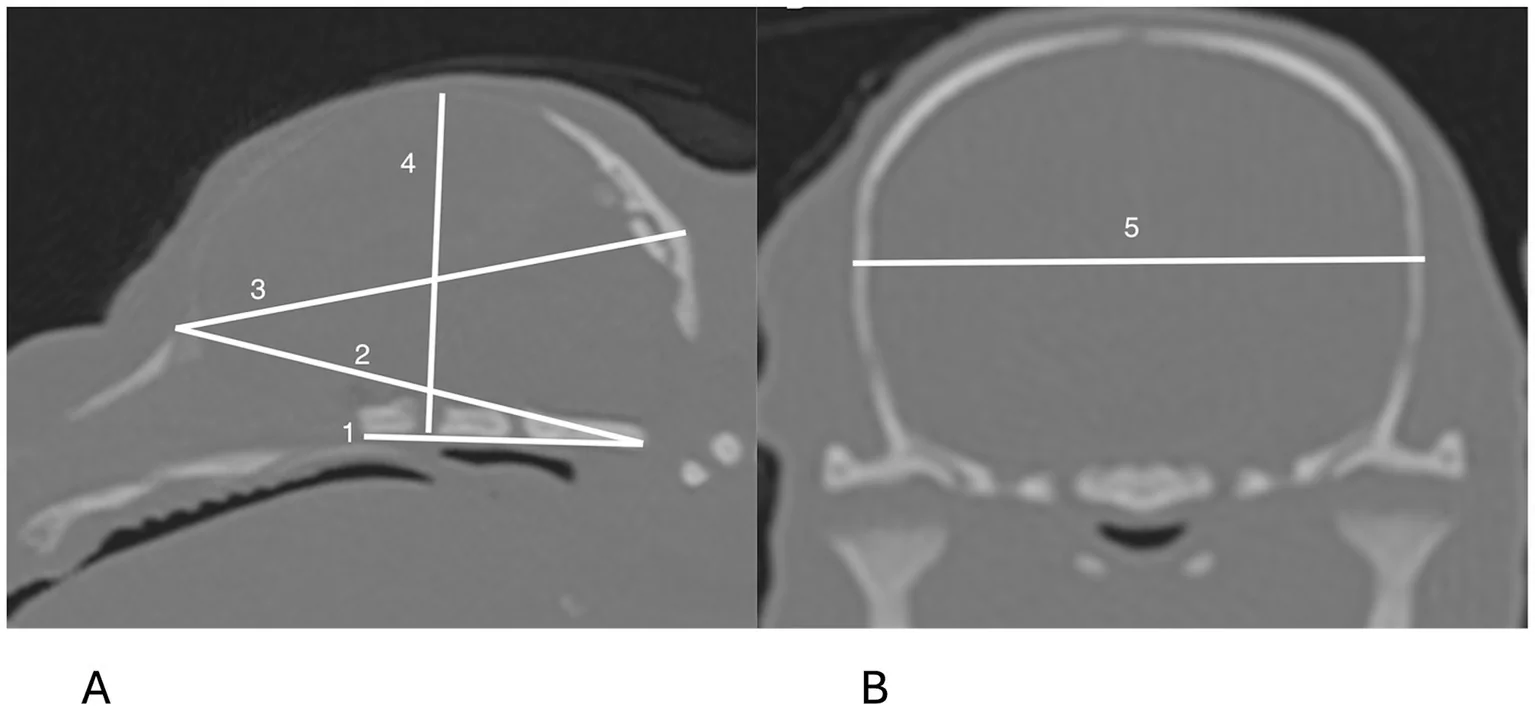
**(A,B)** Craniometric measurements illustrated on mid-sagittal **(A)** and transverse **(B)** CT images of the cranium. (1) Cranial base length 1, (2) Cranial base length 2, (3) Cranial length, (4) Cranial height, (5) Cranial width. Modified from Rautala et al. (15).

Craniometric measurements:

1. *Cranial base length 1*: The length was measured from the basion to the rostral border of the presphenoid bone along the ventral border of the cranial base. In case the presphenoid bone was not fused at midline, the cranial base length 1 was measured slightly parasagittally instead of at midline.
2. *Cranial base length 2*: The length was measured from the basion to the caudal tip of the nasal bone. Due to the small size of the puppies and the open cranial sutures at midline, the caudoventral tip of the frontal bone was not visible in all puppies. Hence, the cranial base length 2 measurement was extended rostrally to the caudal tip of the nasal bone.
3. *Cranial length*: The length was measured from the nasion to the inion. If the inion was poorly defined, the caudal point was defined as the most caudal edge of the cranium.
4. *Cranial height*: The cranial height comprised the maximum height of the cranium perpendicular to the cranial base at the level of the measurement.
5. *Cranial width*: The cranial width comprised the maximum width of the cranium.

The following ratios were calculated to determine the cranial shape:

1. Cranial height to cranial base length 1
2. Cranial height to cranial base length 2
3. Cranial height to length
4. Cranial height to width
5. Cranial index (CrI): $\text{cranial}\frac{\text{width}}{\text{length}}\;x\;100$

### 2.5 Micro-computed tomography analysis

Micro-CT images of the head were used to evaluate the occurrence of cranial fontanelles and other bone defects of the cranium. If a bregmatic fontanelle was visible, the relative width of the bregmatic fontanelle was determined by dividing the bregmatic fontanelle width with the cranial width. Image analysis was conducted from anonymized images with windowing, allowing visualization of the bone by two assessors (ER and A-MK) independently and blinded to each other’s and other diagnostic findings.

The presence, number, and location of cranial fontanelles at cranial sutures and their intersections were evaluated from micro-CT images, as previously described for CT images (1). A fontanelle was defined as a local hypoattenuating area, indicating complete absence of bone at a cranial suture. To differentiate an open cranial suture from a fontanelle, the hypoattenuating area had to be wider than the regional cranial suture(s). As the size of the puppies varied, no specific threshold measure could be used for definition of fontanelle, and the decision was based on each evaluator’s subjective assessment. Lack of bone located at sites other than cranial sutures was recorded as other bone defects. In case of disagreement, the assessors re-evaluated the study together to reach a consensus.

Bregmatic fontanelle and cranial widths were measured from micro-CT images. Due to open cranial sutures, clear demarcation of the fontanelle area was impossible, hence, we used the maximum width to compare the bregmatic fontanelle size. Because the overall size of the puppies varied, instead of absolute fontanelle width, we used the relative width of the bregmatic fontanelle for comparison. During bregmatic fontanelle width measurement, the index lines were positioned tangentially to the cranial surface in sagittal and transverse images and along the midline in dorsal images (Figures 2A–C). As bregmatic fontanelles may extend over the curved surface of the cranium, a maximum intensity projection technique (MIP) was applied for the measurement similar to that previously described (1). A slice thickness of 12–16 mm was chosen for optimal visualization of the fontanelle and to conduct the fontanelle width measurement. Fontanelle width was measured at its widest point. If the fontanelle was equally wide between the frontal and parietal bones, the width between the frontal bones was measured (Figure 2D). Cranial width was measured from transverse images from the widest part of the cranium (15). The mean values from two assessors were used to calculate the relative width of the bregmatic fontanelle.

**Figure 2:**
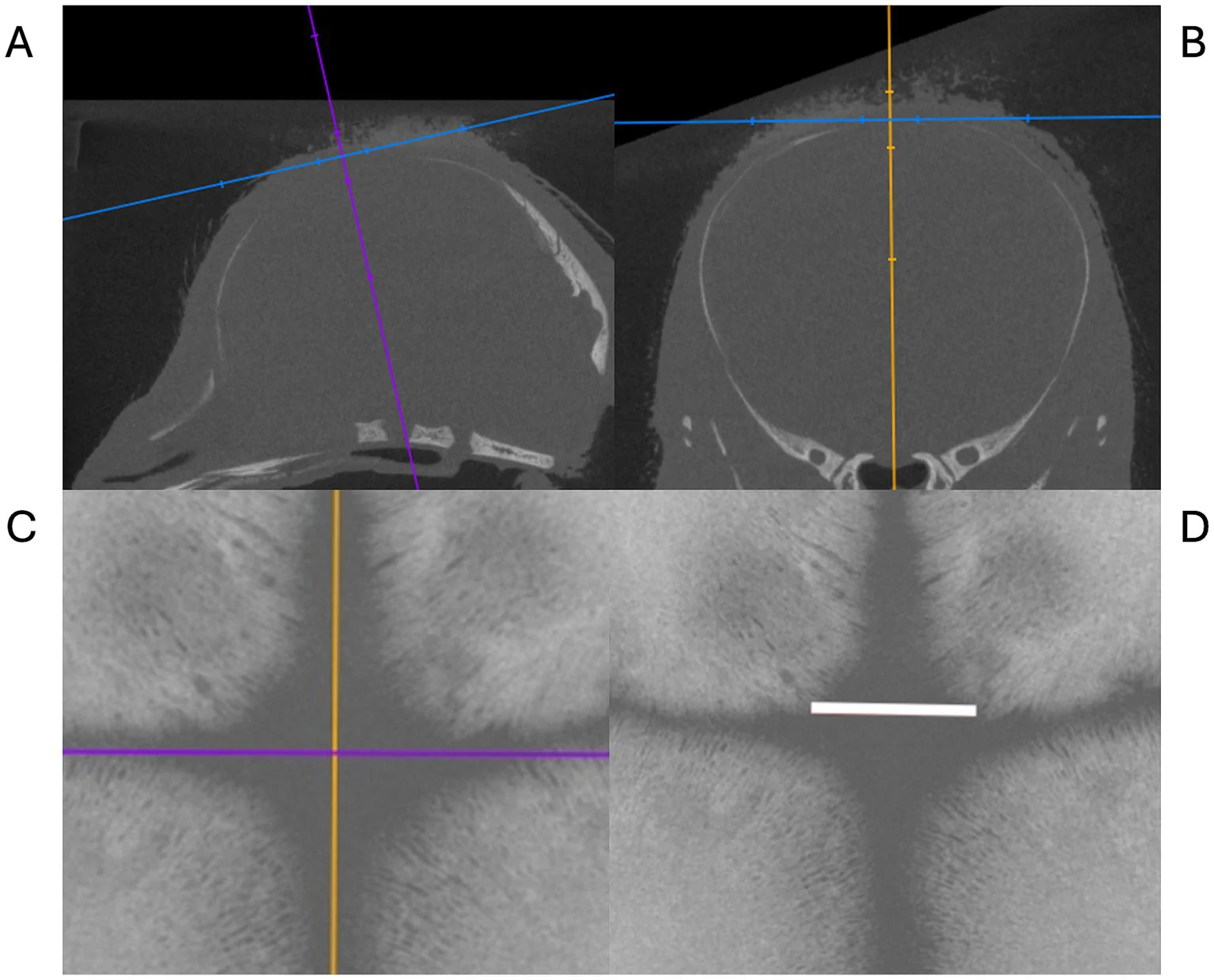
**(A–D)** Micro-CT images in mid-sagittal **(A)**, transverse **(B),** and dorsal **(C,D)** planes illustrating the measurement of the bregmatic fontanelle width: **(A)** For measurement of bregmatic fontanelle width, the index lines were positioned tangentially to bregmatic fontanelle in sagittal image, **(B)** in transverse image, and **(C)** along the midline in dorsal image, **(D)** bregmatic fontanelle width measured from frontal bone to frontal bone in dorsal image.

### 2.6 Necropsy and histological analysis

Necropsy was performed by the veterinary pathologist (SM) for all puppies to evaluate the cause of death and to rule out extracranial diseases that could have affected cranial development. Histological samples of the cranium were used to confirm the presence of cranial fontanelles observed in micro-CT images. Before necropsy, all except three puppies were stored refrigerated for 1–8 days. Three puppies had been stored long term as frozen between death and necropsy (23 days, 29 days, and 6 years 4 months). After the necropsy examination, the entire heads of the puppies were stored in formalin. Before histological sampling, soft tissues were removed from the cranium. Samples were taken in all puppies at predetermined locations of the cranium. The predetermined sampling sites included the bregmatic fontanelle and four specific intersections of the cranial sutures, two on each side of the cranium. These intersections included the intersection of fronto-parietal, spheno-frontal, and spheno-parietal sutures (“sphenoid site”) and the intersection of squamous, occipito-parietal, and occipito-squamous sutures (“mastoid site”) (Figures 3A,C). To collect these samples, the cranium was cut in two blocks using a corundum sawblade (Proxxon Micromot System) (Figures 3A–C). Additionally, if cranial fontanelles were observed either during image analysis (micro-CT) or during macroscopic assessment in the course of sample preparation, these fontanelles were sampled separately. If other fontanelles or bone defects were present, the sample was cut across the bone defect at its largest diameter evaluated by visual inspection. Predetermined cranial suture intersections are common sites of persistent fontanelles in adult Chihuahuas (1) and represent the sphenoid and mastoid fontanelle sites in children (5), hence being chosen for sampling.

**Figure 3:**
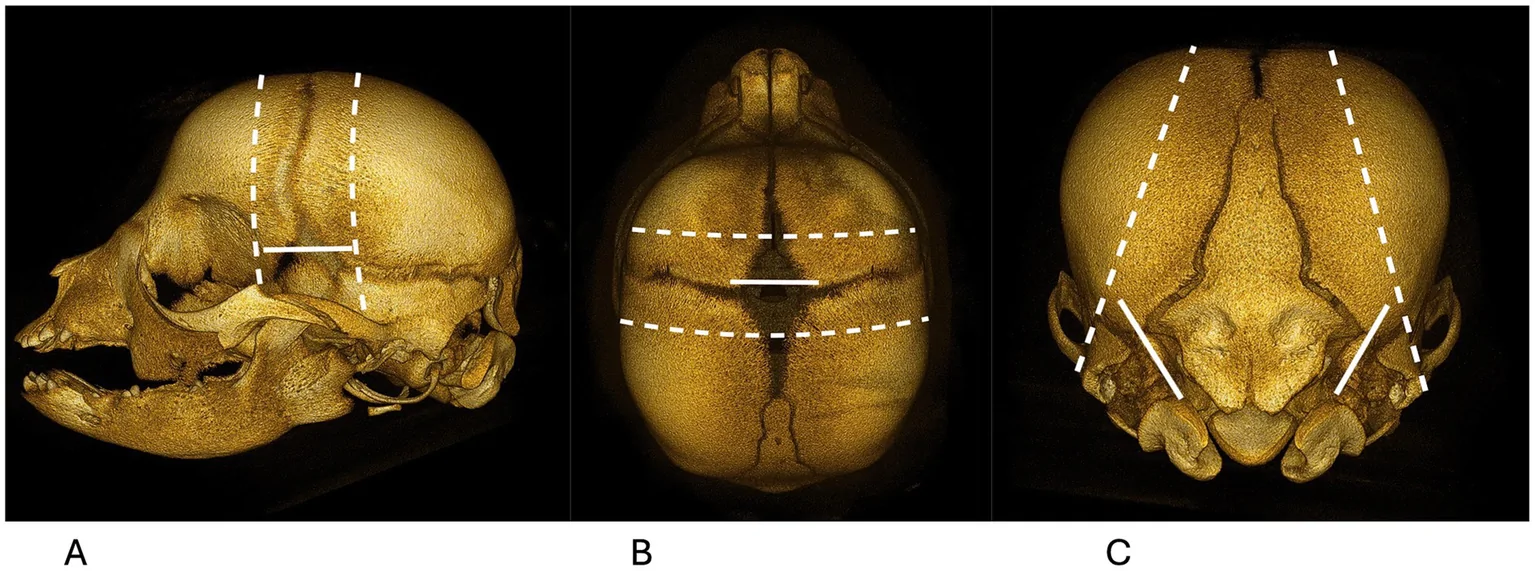
**(A–C)** Histological sampling of the cranium: **(A)** A block cut from the cranium, including bregmatic fontanelle site, bilateral fronto-parietal sutures, and intersection of fronto-parietal, spheno-frontal, and spheno-parietal sutures (between dotted lines), with sample site of the cranial suture intersection (“sphenoid site”) marked by a solid line. **(B)** Sample site of bregmatic fontanelle marked by a solid line. **(C)** A block cut from the cranium, including sagittal suture, bilateral parieto-interparietal and occipito-parietal sutures, and intersection of squamous, occipito-parietal, and occipito-squamous sutures (between dotted lines), with sample site of the cranial suture intersection (“mastoid site”) marked by a solid line.

Before the histological analysis, the cranial samples were decalcified with 0.5 M ethylenediaminetetraacetic acid (EDTA) for 3–6 days at pH 7.5. The samples were embedded with paraffin, cut at 4 μm sections, and stained with hematoxylin and eosin. Veterinary pathologist (SM) evaluated all histological samples for occurrence of cranial fontanelles and other bone defects. Histological samples were used to confirm the presence of cranial fontanelles observed in micro-CT images and to differentiate cranial fontanelles from cranial sutures. In histological analysis, a fontanelle was defined as an area of continuous fibrous tissue at the cranial suture or intersection of cranial sutures that is wider than the corresponding suture(s) in the area and has no bone or bone islands. A cranial suture was defined as a narrow band of connective tissue located between cranial bones (19). Other bone defect of the cranium was defined as an area of continuous fibrous tissue located elsewhere in the bone than at the cranial suture. The pathologist was blinded to the signalment, and micro-CT findings of the puppies while performing the histological analysis of the cranial fontanelles. If there was a discrepancy in fontanelle occurrence between the micro-CT analysis and the histological samples, histology was considered the definitive result.

The level of autolysis was evaluated in each puppy macroscopically at the time of histological sample collection and during histological analysis. If the skull or histological samples showed autolytic changes that would increase the fragility of the skull or prevent detection of bone and fibrous tissue in histological analysis, the specimen was excluded from the study.

### 2.7 Statistical analysis

Duration of gestation, age at death, sex, and mode of delivery were compared between the groups. Minimum duration of gestation and age at the time of death were compared between Chihuahuas and non-brachycephalic puppies by using Wilcoxon-Mann–Whitney test. Because some dogs had several matings, minimum duration of gestation calculated from the last mating was used for statistical analysis. Sex and mode of delivery were compared between Chihuahuas and non-brachycephalic puppies using chi-square test.

Descriptive statistics were calculated for the craniometric measurements. Data are presented as mean (± standard deviation (SD) and range). The differences in craniometric ratios between Chihuahuas and non-brachycephalic puppies were analyzed with independent samples t-tests / Welch’s test depending on the result of the test of equal variances, each craniometric ratio separately.

Micro-CT findings were combined with histological samples of the cranium, and puppies with histologically confirmed results of the cranial fontanelles were included in the analysis of cranial fontanelles. The occurrence of cranial fontanelles was compared between Chihuahuas and non-brachycephalic puppies by calculating risk difference, together with 95% exact confidence intervals, accompanied by Fisher’s exact tests.

The bregmatic fontanelle width to cranial width ratio was compared between Chihuahuas and non-brachycephalic puppies with independent samples t-tests. To evaluate the repeatability of the measurement methods, the inter-rater reliabilities were assessed separately for bregmatic fontanelle width and cranial width measured from micro-CT images. A repeatability statistic between the assessors was calculated from a one-way analysis of variance model, where the effect of the puppy served as the sole fixed effect. In these models, the within-group variation describes the variation between assessors. These repeatability values can be considered as a percentage of perfect agreement. Intraclass correlation coefficients (ICC) were calculated to assess the consistency between the evaluators. Values < 0.5 indicate poor reliability, values between 0.5 and 0.75 moderate reliability, values between 0.75 and 0.9 good reliability, and values > 0.9 excellent reliability (20).

The association of relative width of the bregmatic fontanelle with cranial dimensions was analyzed using linear regression and included only puppies with a bregmatic fontanelle. The relative width of the bregmatic fontanelle served as the response; each cranial dimension was assessed separately and used as the explanatory term together with puppy group which was included in the models as a covariate. The normality of the model residuals was investigated using Shapiro–Wilk tests and normal QQ-plots. Influential observations were investigated by calculating Cook’s distances for all observations and applying conventional > 1 as the threshold to define highly influential cases. No observations exceeded this threshold.

Due to the exploratory nature of the study, no multiplicity adjustments were used. The results of the conducted statistical analyses were used as hypothesis-generating rather than confirmatory*. p*-values of < 0.05 were considered statistically significant. All *p*-values are 2-sided, not adjusted for multiple testing. All statistical analyses were done using the SAS System for Windows, version 9.4 (SAS Institute Inc., Cary, NC, United States).

## 3 Results

In total, 57 puppies were collected for the study. Seven puppies were excluded from the study for various reasons. One puppy was excluded because its age exceeded the inclusion criteria (age at time of death 8 days). Three Staffordshire bullterriers were excluded because they were considered as brachycephalic puppies. After imaging studies and necropsy, two more puppies were excluded due to cleft palate and hydrocephalus observed in CT in one puppy and cheilochisis in one puppy. Furthermore, one puppy that had been stored as frozen for 29 days was excluded because of extensive postmortem autolytic changes causing fragility of the cranium during histological sample collection. No space-occupying lesions of the brain were detected in puppies that underwent MRI examination.

The study included 24 Chihuahuas and 26 non-brachycephalic puppies. Chihuahuas originated from 21 litters; in two occasions we included more than one Chihuahua puppy from the same litter, two puppies from one and three puppies from another litter. Breeds in the non-brachycephalic group were as follows: Collie (*n* = 9), Labrador retriever (*n* = 6), German shepherd (*n* = 3), Toy poodle (*n* = 1), Lagotto Romagnolo (*n* = 1), Great Dane (*n* = 1), Norfolk terrier (*n* = 1), Scottish terrier (*n* = 1), Fox terrier (*n* = 1), Bernese Mountain dog (*n* = 1), and Parson Russell terrier (*n* = 1). In non-brachycephalic group, puppies originated from 18 different litters: three long haired collies, two Labrador retrievers, two German shepherds, and five short haired collies came from the same litter.

There was no significant difference in the age at death, gender distribution or mode of delivery between the groups (Table 1). Of the puppies, 25 were females (13 Chihuahuas and 12 non-brachycephalic puppies) and 23 males (10 Chihuahuas and 13 non-brachycephalic puppies). For two puppies, the sex was not recorded. The mean minimum duration of gestation was shorter in Chihuahuas than in non-brachycephalic puppies (60.2 days, range 58–63, and 62.0 days, range 58–66, respectively, *p* < 0.0001; Table 1). Baseline information of each puppy is provided in the Supplementary Table 1.

**Table 1:** Comparison of minimum duration of gestation, age, gender and mode of delivery between Chihuahuas and non-brachycephalic puppies.

Variable	Statistics	Chihuahuas	Non-brachycephalic puppies	Overall	*p*-value
Minimum duration of gestation (days)	Mean (SD)	60.2 (1.25)	62.0 (1.83)	61.1 (1.81)	<0.0001
	Median	60.0	62.0	61.0	
	Range	58–63	58–66	58–66	
	n	24	26	50	
Age (days)	Mean (SD)	0.4 (1.28)	0.5 (1.21)	0.4 (1.23)	0.7720
	Median	0.0	0.0	0.0	
	Range	0–6	0–4	0–6	
	n	24	26	50	
Gender	nk	1	1	2	0.5549
	Female	13	12	25	
	Male	10	13	23	
Mode of delivery	nk	1	0	1	0.0556
	Cesarean section	7	15	22	
	Natural	16	11	27	

*p*-values < 0.05 were considered statistically significant. SD, standard deviation; nk, not known.

The postmortem storage conditions are described for each included puppy in Supplementary Table 1. Because the bregmatic fontanelle sample of one non-brachycephalic puppy failed due to autolytic changes, this puppy was excluded from the analyses of cranial fontanelles and relative width of the bregmatic fontanelle (Figure 4). In the rest of the included puppies, autolytic changes were not considered to affect histological analysis of cranial fontanelles or the craniometric or bregmatic fontanelle width measurements.

**Figure 4:**
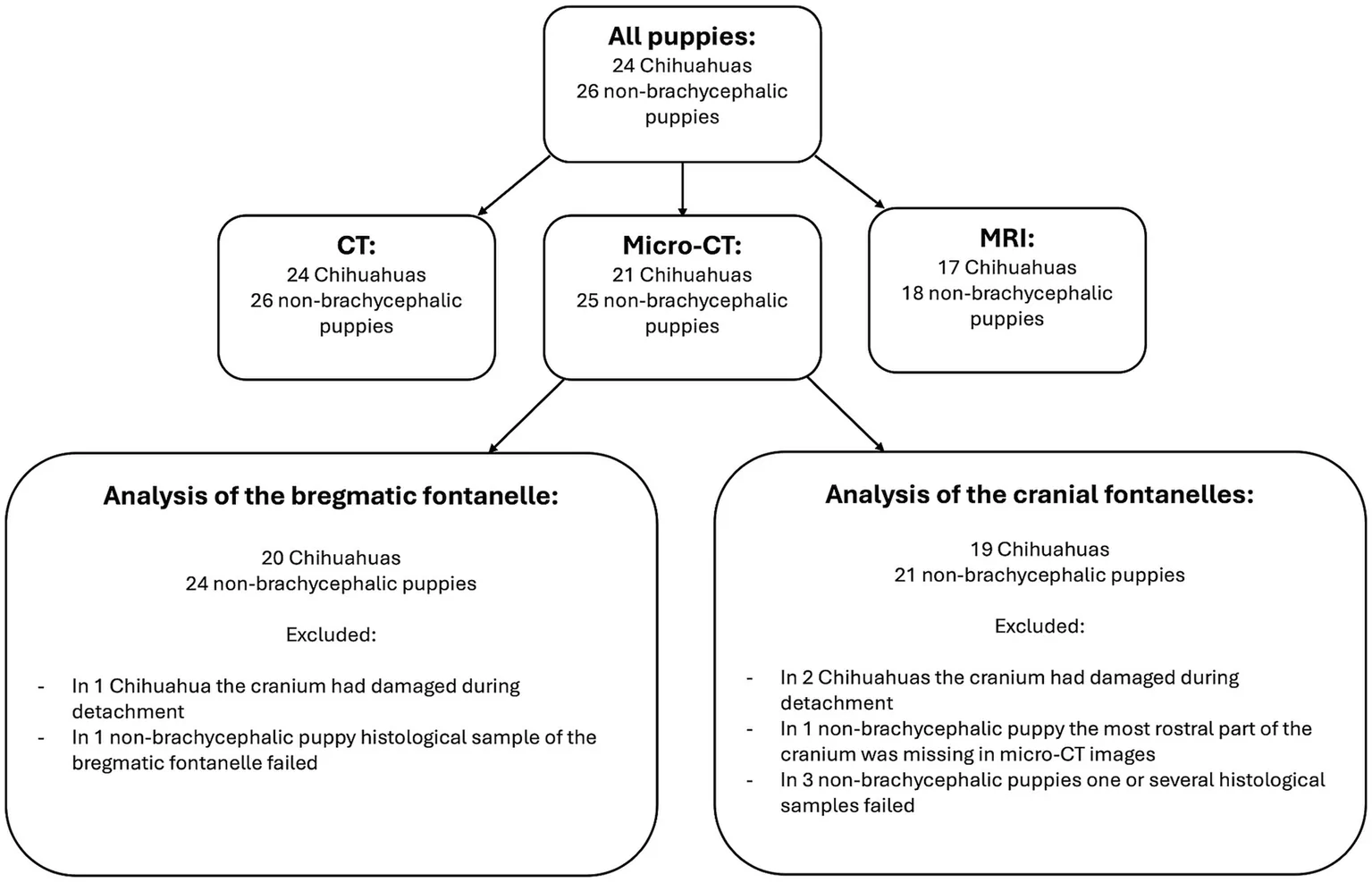
Numbers of puppies participating in the imaging studies and puppies excluded from the micro-CT analysis of the bregmatic fontanelle and cranial fontanelles.

The numbers of puppies participating in the imaging studies and puppies excluded from the micro-CT analysis of bregmatic fontanelle and cranial fontanelles are presented in Figure 4.

### 3.1 Craniometric ratios in Chihuahuas and non-brachycephalic puppies

The mean values (± SD, range) of the craniometric ratios of the Chihuahuas and non-brachycephalic puppies are presented in Table 2. The mean value for CrI was 86.6 (2.33, 81.8–91.9) for Chihuahuas and 75.6 (4.53, 66.7–83.2) for non-brachycephalic puppies (Table 2). Cranial height to cranial base length 1 and 2 ratios, cranial height to length ratio, and CrI were significantly higher in Chihuahuas than in non-brachycephalic puppies (*p* < 0.001 for each analysis) (Table 3). For the Chihuahua puppy with three cranial fontanelles, cranial height to cranial base length 2 ratio was 0.87, and cranial height to length ratio 0.78.

**Table 2:** Mean craniometric ratios of Chihuahuas and non-brachycephalic puppies.

Ratio	Chihuahuas (24)			Non-brachycephalic puppies (26)		
	Mean	SD	Range	Mean	SD	Range
Cranial height/cranial base length 1	1.47	0.091	1.26–1.63	1.13	0.104	1.00–1.43
Cranial height/cranial base length 2	0.82	0.032	0.76–0.88	0.67	0.052	0.59–0.77
Cranial height/length	0.73	0.024	0.69–0.78	0.63	0.041	0.58–0.70
Cranial height/width	0.84	0.024	0.80–0.89	0.83	0.027	0.78–0.88
Cranial index (CrI)	86.6	2.33	81.8–91.9	75.6	4.53	66.7–83.2

CrI, cranial index; SD, standard deviation.

**Table 3:** Differences in craniometric ratios between Chihuahuas (24) and non-brachycephalic puppies (26).

Response	Estimate	SEM	95% CI	*p*-value
Cranial height/cranial base length 1	0.3362	0.0276	0.2807–0.3918	<0.0001
Cranial height/cranial base length 2	0.1517	0.0122	0.1275–0.1760	<0.0001
Cranial height/length	0.1017	0.0097	0.0826–0.1208	<0.0001
Cranial height/width	0.0124	0.0073	−0.0022–0.0270	0.0946
Cranial index (CrI)	10.9998	1.0318	8.9601–13.0395	<0.0001

*p*-values < 0.05 were considered statistically significant. CrI, cranial index; SEM, standard error of the mean; CI, confidence interval.

### 3.2 Analysis of cranial fontanelles and other bone defects

Micro-CT imaging was available for 21 Chihuahuas and 25 non-brachycephalic puppies. In two Chihuahuas, crania had damaged during the detachment; in one Chihuahua the damage prevented histological sampling of the bregmatic fontanelle and the mastoid sites, and this puppy was excluded from the analysis on cranial fontanelles and the analysis of bregmatic fontanelle. In one Chihuahua the damage was limited to the caudal part of the cranium and prevented the histological sampling of the mastoid site. Hence, this puppy was excluded from the analysis of cranial fontanelles, but it was included in the analysis of bregmatic fontanelle. In one non-brachycephalic puppy, the most rostral part of the cranium was missing in micro-CT imaging. Furthermore, in two non-brachycephalic puppies one or both histological samples of the mastoid site failed. These three puppies were excluded from the analysis of cranial fontanelles. Finally, in one non-brachycephalic puppy the histological sample of the bregmatic fontanelle failed, and this puppy was excluded from the analysis of the cranial fontanelles and the analysis of bregmatic fontanelle. (Figure 4).

Analysis of cranial fontanelles was based on micro-CT images of 40 puppies including 19 Chihuahuas and 21 non-brachycephalic puppies (Figure 4). Of these, 25 puppies had cranial fontanelles: 18 Chihuahuas and 6 non-brachycephalic puppies had one cranial fontanelle, which was the bregmatic fontanelle (Figures 5A,B). Additionally, one Chihuahua had three fontanelles, including the bregmatic fontanelle and two symmetrical fontanelles bilaterally in the frontoparietal cranial suture (Figure 5C). The proportion of puppies having at least one cranial fontanelle was significantly higher in Chihuahuas than in non-brachycephalic puppies (*p* < 0.001, risk difference 71.4, 95% CI 47.6–88.8%). One Chihuahua had other bone defect located at the right frontal bone.

**Figure 5:**
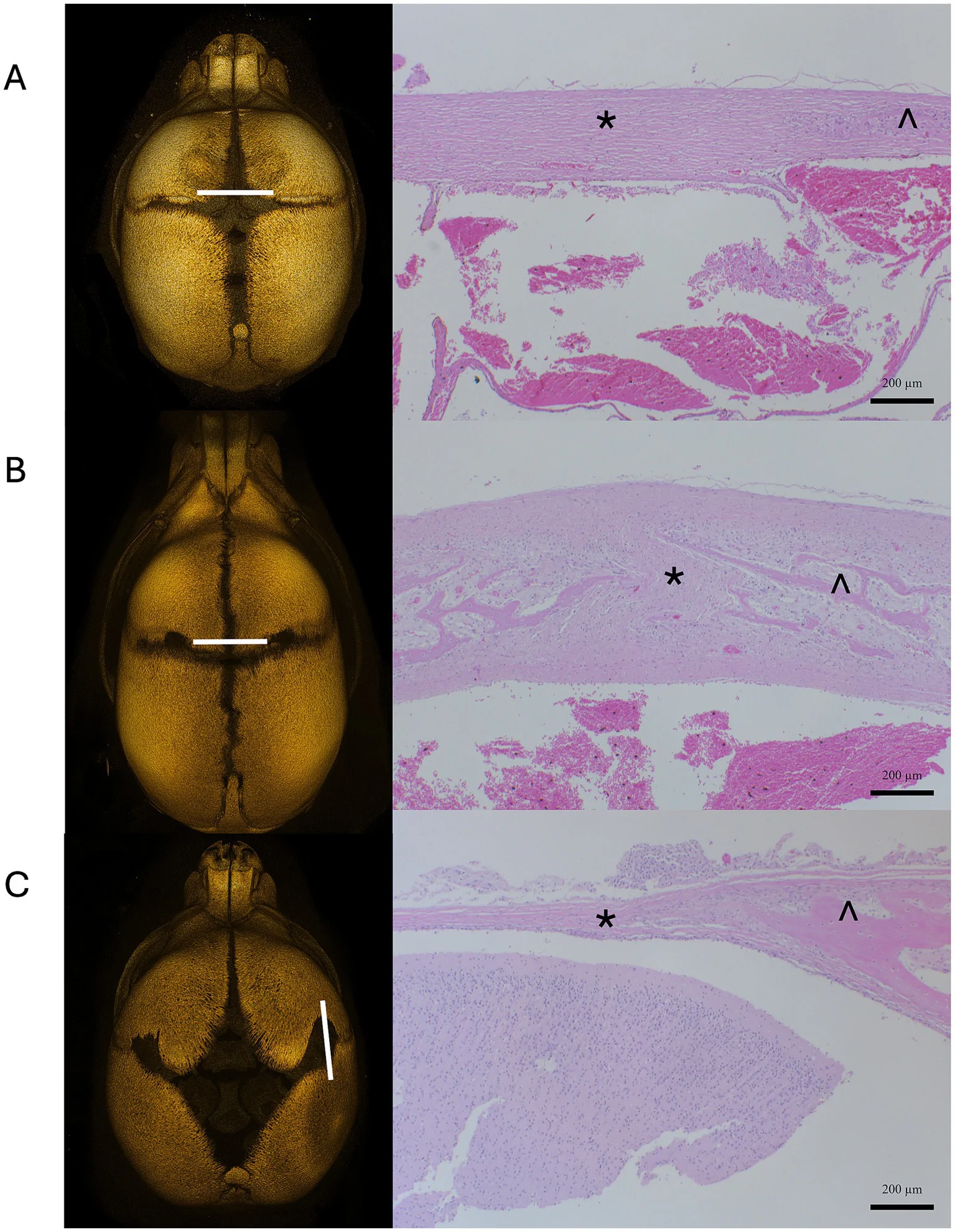
**(A–C)** Three-dimensional volumetric reconstructions of micro-CT images of the cranial fontanelle sites and corresponding histological sections acquired with 5 × objective stained with hematoxylin and eosin; the site of the histological sample is marked with a solid line. **(A)** Chihuahua puppy with bregmatic fontanelle. **(B)** Collie puppy showing intersection of cranial sutures at bregma not considered as bregmatic fontanelle. **(C)** Chihuahua puppy with bilateral fontanelles in fronto-parietal sutures. In histological samples *indicates fibrous tissue and ^ bone tissue in cranial suture or fontanelle.

In all dogs, the mastoid fontanelle site showed a hypoattenuated area in the micro-CT images. However, histological examination showed a thick cartilage, areas of ossification, and an oblique fibrous structure resembling a suture in this area, and hence, it was not considered a fontanelle.

Altogether 880 cranial sutures or intersections of cranial sutures were evaluated in 19 Chihuahuas and 21 non-brachycephalic puppies. Disagreement regarding detection of fontanelles between the evaluators occurred in 13/880 sutures or intersections of cranial sutures (1,48%). Disagreement in fontanelle detection occurred in six Chihuahuas and represented 2,15% of all sutures and intersections evaluated in Chihuahuas, and in four non-brachycephalic puppies and represented 0,91% of all sutures and intersections evaluated in non-brachycephalic puppies. In all cases disagreement involved distinction of a small fontanelle from a cranial suture.

In eight puppies, there was disagreement between the micro-CT and histological analysis. Narrow bregmatic fontanelle was observed in micro-CT in four non-brachycephalic puppies; however, the histological sample was interpreted as a cranial suture. Furthermore, in micro-CT analysis, a small fontanelle was observed in the interfrontal suture in one Chihuahua puppy, in the fronto-parietal suture in one Chihuahua puppy, and in the interfrontal suture and parieto-interparietal suture in one Chihuahua puppy. However, these fontanelles were not detected during histological sampling of the cranium, and hence, they were not considered as cranial fontanelles in the statistical analysis. Finally, in one Chihuahua puppy, ossification defects were observed bilaterally in frontal bones in micro-CT. In histology, continuous fibrous tissue was observed between the bone ends on the right side, whereas a continuous thin layer of bone was observed on the left side. Hence, only the right side was interpreted as other bone defect.

### 3.3 Relative width of the bregmatic fontanelle and its association with craniometric ratios

The relative width of the bregmatic fontanelle was determined for 44 puppies including 20 Chihuahuas and 24 non-brachycephalic puppies. The relative width of the bregmatic fontanelle was significantly larger in Chihuahuas than in non-brachycephalic puppies (estimate 0.1333, SEM 0.0365, 95% CI 0.0580–0.2087, *p* = 0.0013). Inter-rater reliability of measurements of the cranial width and bregmatic fontanelle width from micro-CT images was > 99% and ICC values were > 0.98 (Table 4).

**Table 4:** Inter-rater repeatability of micro-CT measurements between the 2 assessors.

Repeatability percentage (one-way ANOVA)				Intraclass correlation coefficient (ICC)			
Variable	Dogs	Residuals	Inter-rater reliability	Type	ICC	Lower bound	Upper bound
Cranial width	249.172	2.385	99.052	ICC1	0.9975	0.9944	0.9989
Bregmatic fontanelle width	792.964	1.391	99.825	ICC1	0.9817	0.9602	0.9917

The association of the relative width of the bregmatic fontanelle with craniometric ratios was determined in 20 Chihuahuas and 6 non-brachycephalic puppies that had a bregmatic fontanelle. The relative width of the bregmatic fontanelle was larger in puppies with higher cranial height to cranial base length 2 and cranial height to length ratios (*p* = 0.022 and *p* = 0.023, respectively; Table 5).

**Table 5:** Association of relative bregmatic fontanelle width with craniometric ratios in Chihuahuas (20) and non-brachycephalic puppies (6).

Model	R2	Variable	Estimate	Lower 95% CI	Upper 95% CI	*p*-value
Cranial height/cranial base length 1	0.43	Chihuahua	0.063	−0.046	0.173	0.244
		Cranial height/cranial base length 1	0.282	−0.050	0.613	0.092
Cranial height/cranial base length 2	0.49	Chihuahua	0.029	−0.083	0.141	0.601
		Cranial height/cranial base length 2	1.027	0.160	1.894	0.022
Cranial height/length	0.49	Chihuahua	0.058	−0.036	0.152	0.215
		Cranial height/length	1.350	0.205	2.495	0.023
Cranial height/width	0.41	Chihuahua	0.140	0.066	0.215	< 0.001
		Cranial height/width	1.002	−0.452	2.456	0.167
Cranial index (CrI)	0.41	Chihuahua	0.075	−0.034	0.184	0.166
		Cranial index	0.008	−0.003	0.019	0.150

*p*-values < 0.05 were considered statistically significant. CrI, cranial index; CI, confidence interval.

## 4 Discussion

Bregmatic fontanelle occurred more frequently and was relatively larger in newborn Chihuahuas than in non-brachycephalic puppies. Cranial fontanelles other than bregmatic fontanelle were seldom detected even in Chihuahuas. Based on our results, cranial morphology differs between newborn Chihuahuas and non-brachycephalic puppies, with Chihuahuas having a relatively shorter, wider, and higher cranium than non-brachycephalic puppies. Furthermore, puppies with a shorter and higher cranium, indicating a higher degree of brachycephaly, had a relatively wider bregmatic fontanelle.

### 4.1 Cranial fontanelles in newborn Chihuahuas and non-brachycephalic dogs

All but one puppy had either none or only one cranial fontanelle, which was the bregmatic fontanelle. Our findings correspond to the description of cranial fontanelles in the literature, as bregmatic fontanelle is the only fontanelle reported in puppies and it may normally be either open or closed at birth (3, 4, 21).

We detected a significant difference in occurrence of bregmatic fontanelle between newborn Chihuahuas and non-brachycephalic puppies, which suggests that differences in occurrence of bregmatic fontanelle exist between these two groups during the fetal period. Accordingly, variation in the occurrence of bregmatic fontanelle at the time of birth in dogs has been observed in previous studies. In beagle dogs, bregmatic fontanelle is reported to close before birth (3). However, in studies including mixed breed and beagle dogs, ultrasound of the brain through the bregma has been possible up to 4 weeks after birth, suggesting closure of the bregmatic fontanelle within 1 month of age (4, 21). In Chihuahuas, open bregmatic fontanelle (molera) has long been a desired trait, and it is still accepted in some breeding guidelines (22). Favoring occurrence of bregmatic fontanelle in the breed causes strong genetic selection, which could possibly affect the cranial ossification already during the fetal period and explain differences in occurrence of bregmatic fontanelle between newborn Chihuahuas and non-brachycephalic puppies.

Due to the high number of persistent fontanelles located outside the bregma in adult dogs, we expected to observe cranial fontanelles other than bregmatic fontanelle also in newborn Chihuahuas. However, cranial fontanelles at locations other than the bregma or other bone defects were seldom present. In adult small-sized dogs, persistent fontanelles are detected in all surfaces of the cranium (1, 7). In adult Chihuahuas, bregmatic fontanelle is the most common persistent fontanelle, however, approximately 50% of persistent fontanelles occur at locations other than the bregma (1). Furthermore, in one study cohort, 82.9% of adult toy-sized dogs of different breeds had osseus defects of the cranium outside the bregma, with the caudal part of the cranium being the predominant site for defects (7). The difference between newborn and adult Chihuahuas might suggest that the bone defects observed in adult Chihuahuas develop during postnatal cranial growth. Postnatal bone remodeling and resorption are possible mechanisms affecting development of persistent fontanelles and this hypothesis is indirectly supported by our findings. However, longitudinal imaging and histological studies of Chihuahua puppies at different ages are needed to confirm this theory.

Final sample size used for the analysis of cranial fontanelles was below the planned sample size. However, the results were unexpected as only one Chihuahua had cranial fontanelles located outside bregma. Hence, the occurrence of cranial fontanelles located outside bregma could not be compared between Chihuahuas and non-brachycephalic puppies and the initial sample size calculation was no longer appropriate. Approximately half of the persistent fontanelles in adult Chihuahuas are located outside bregma (1). Hence, it is likely that if all persistent fontanelles observed in adult Chihuahuas were congenital bone defects we would have seen more of them in these newborn puppies. However, our results need be confirmed in a larger study population.

In all puppies, the mastoid fontanelle site showed lack of bone in micro-CT images, but histology revealed cartilage and ongoing ossification in this area. This finding was uniform in both Chihuahuas and non-brachycephalic puppies and most likely represents normal cranial ossification. In the mastoid area, supraoccipital and exoccipital bones forming the intersection with the adjacent temporal and parietal bones ossify via endochondral ossification, whereas temporal and parietal bones ossify through intramembranous ossification (3, 23). Both cartilage and fibrous tissue appear hypoattenuated in CT images. Hence, histological samples are necessary to confirm cranial fontanelles at this site in puppies.

One Chihuahua had other bone defect dorsally in the frontal bone. The cause of this lesion is unknown. One explanation might be erosion of bone due to pressure exerted by the brain. In children, foramina of the frontal bones (frontal foramina) occur rarely with Chiari type II malformation and congenital hydrocephalus, but the etiology of the frontal foramina is unclear (24). Defects of the frontal bones have also been reported in children as an isolated finding without evidence of neural tissue abnormalities, possibly representing partial aplasia of the frontal bones (25, 26). However, these defects are often large and extend to the midline, hence differing from the lesion observed in our study.

### 4.2 Craniometric ratios in Chihuahuas and non-brachycephalic dogs

Cranial height to cranial base length 1 and 2 ratios, cranial height to length ratio, and CrI were greater in Chihuahuas than in non-brachycephalic puppies. This indicates that Chihuahuas have a relatively shorter, wider, and higher cranium than non-brachycephalic puppies. Our results suggest that cranial growth differs between Chihuahuas and non-brachycephalic puppies already during the fetal period. Previous information regarding cranial shape in newborn puppies is limited and partly contradictory. Similar to our study, Andreis et al. (27) showed that skull index and anatomically defined CrI can differentiate between newborn brachycephalic and non-brachycepahlic puppies of different breeds. However, radiographically determined CrI could not separate the two groups (27). The discrepancy between the results for CrI in previous and the current study could be related to breed distribution, as Chihuahua was the only brachycephalic breed included in our study. Breeds generally considered brachycephalic can have variation in cranial shape (10, 28), some brachycephalic dogs, such as Boxers, having a narrow and high cranium instead of a short and wide cranium (28). Hence, the breeds included in the study cohort might influence the results. Furthermore, different imaging modalities used for craniometric measurements might have affected the results.

It is currently believed that development of brachycephaly in dogs is associated with premature closure of the cranial base synchondroses and premature closure (craniosynostosis) of one or multiple cranial sutures (13, 28, 29). Brachycephalic dogs have greater number of closed cranial sutures than mesaticephalic or dolichocephalic dogs (28, 29). However, studies comparing cranial suture closure in newborn brachycephalic and non-brachycephalic puppies are lacking. Bilateral fronto-parietal craniosynostosis occurs in extremely brachycephalic peke-face Persian cats, and this condition is observed already in neonates (30). Similarly, bilateral craniosynostosis of the fronto-parietal sutures causing brachycephalic cranial shape in children is typically an intrauterine event that can be detected perinatally (31, 32). Future studies investigating cranial suture and synchondrosis status in newborn Chihuahuas are warranted to determine whether craniosynostosis explains the differences in cranial shape between Chihuahuas and non-brachycephalic puppies.

Interestingly, the mean cranial height to length and height to width ratios and CrI of the newborn Chihuahuas in this study were close to the mean values observed in a population of adult Chihuahuas: cranial height to length ratio being 0.73 in newborns and 0.71 in adults, cranial height to width ratio 0.84 in newborns and 0.86 in adults, and CrI 86.6 in newborns and 82.24 in adults (15). This suggests that cranial shape might not change considerably during growth in Chihuahuas. In mesaticephalic beagle dogs, the cranium is expected to grow in the early postnatal period in equal proportions in length and width (33). However, as the cranial growth continues, it might not be uniform in length and width. Onar (9) showed that in German shepherd puppies mean CrI was significantly higher in a group of puppies aged between 45 and 60 days than in puppies aged between 61 and 105 days. This suggests that as the puppies grow older the length of the cranium increases more than the width, leading to a decrease in CrI and more dolichocephalic cranial shape (9). Our results suggest that in Chihuahuas restricted longitudinal cranial growth and consecutive compensatory growth in cranial width and height occur already during the fetal period. Hence, it seems possible that during the intense postnatal growth, the brain needs to adjust to the relatively restricted cranial shape. In dogs, rapid growth of the brain occurs during the first 2–3 months of life. During this time the brain reaches 50–75% of its adult size (34). The growing brain shapes the cranium, which might cause resorption of the cranial bones and possibly lead to the formation of cranial bone defects in Chihuahuas.

Cranial height to width ratio did not differ between Chihuahuas and non-brachycephalic puppies. In adult dogs, this ratio differs significantly between brachycephalic and mesaticephalic breeds, with brachycephalic dogs having a higher width to height ratio, indicating a relatively wider cranium (28). Hence, although not present at the time of birth, differences in cranial height to width ratio between Chihuahuas and non-brachycephalic breeds might develop during cranial growth.

### 4.3 Relative width of the bregmatic fontanelle and its association with the breed and craniometric ratios

The relative width of the bregmatic fontanelle was larger in newborn Chihuahuas than in non-brachycephalic puppies, suggesting that differences in ossification of the bregmatic fontanelle occur between breeds during the fetal period. Information regarding differences in the relative size of the bregmatic fontanelle between different dog breeds is lacking. In healthy newborn children, the size of the anterior fontanelle (corresponding to the bregmatic fontanelle in dogs) exhibits substantial variation (35). Considerable ethnic variation exists in the normal size of the anterior fontanelle in children, possibly indicating the impact of genetic and geographic components on fontanelle size (36–38).

In dogs, variation of bregmatic fontanelle size in different breeds during prenatal period is unknown. Although the minimum gestational age was shorter in Chihuahuas than in non-brachycephalic puppies, all puppies were considered being full term. Some dogs had several matings which complicated the evaluation of exact gestational age. Hence, the minimum gestational age was used in the statistical analyses, which might have underestimated the duration of gestation in some puppies. The effect of minor difference in gestational age on relative width of the bregmatic fontanelle is unknown. The difference in mean gestational age was only 2 days which is a short period in the overall duration of gestation. Furthermore, the difference in the relative width of the bregmatic fontanelle between Chihuahuas and non-brachycephalic puppies was remarkable, hence the finding is unlikely explained by the difference in gestational age alone.

Larger relative width of the bregmatic fontanelle in newborn Chihuahuas than in non-brachycephalic puppies could be associated with the extremely small size of the Chihuahua breed. In adult Chihuahuas, persistent fontanelles are larger and more numerous in dogs with lower body weight (2). Furthermore, in adult Chihuahuas, higher CrI is associated with lower body weight, indicating increased widening and shortening of the cranium in smaller dogs (15). In general, smaller species have relatively larger brain size than larger species. This can lead to more circular cranial shape and decreased mineralization of the cranium, possibly allowing more efficient accommodation of the brain into the diminished cranium (39, 40). As Chihuahua is one of the smallest dog breeds in the world, a relatively wide bregmatic fontanelle could reflect the disproportion between brain size and the cranial vault and serve to accommodate a relatively large brain into the cranium. A relatively large brain might affect cranial ossification and lead to widening of the bregmatic fontanelle.

Interestingly, abnormally small body size is associated with a large anterior fontanelle in newborn children. In normally developed newborns, both negative and positive correlations of anterior fontanelle size with birth weight have been reported (37, 41, 42). However, in children who are small for gestational age, the size of the anterior fontanelle is larger than in children with body weight appropriate for gestational age (38, 43), possibly due to delayed intrauterine bone maturation (44, 45). Various reasons can result in small body weight for gestational age in children, including factors related to maternal health, placental insufficiency, and fetal genetics (46). Whether delayed intrauterine osseus maturation affects the size of the bregmatic fontanelle in Chihuahuas remains unknown. In children, large anterior fontanelle is also associated with achondroplasia, a skeletal dysplasia causing dwarfism (5). Most cases in humans are caused by a variant in the transmembrane domain of fibroblast growth factor receptor 3 (FGFR3) gene (47, 48). Thus far, a similar variant has not been detected in dogs (49).

The relative width of the bregmatic fontanelle was larger in puppies with relatively higher, and shorter cranium. In adult Chihuahuas, shorter and higher cranium indicating a higher degree of brachycephaly has been associated with more numerous and larger persistent fontanelles (15). Furthermore, an open bregmatic fontanelle has been shown to be associated with cranial shape in adult toy-sized dogs of different breeds (7). Restricted longitudinal cranial growth directs the growing brain in the direction of least resistance and compensatory cranial growth occurs in width and height (14). As cranial ossification is directed by the growing brain, this compensatory growth could increase the pressure exerted by the brain toward the dorsal cranial bones and lead to widening of the bregmatic fontanelle. Interestingly, the only Chihuahua with additional cranial fontanelles in fronto-parietal sutures had cranial height to cranial base length 2 ratio, and cranial height to length ratio in the upper quarter of the Chihuahua group. This could further support our finding that a higher degree of brachycephaly might be associated with dorsal cranial ossification already in newborn Chihuahuas. Enlargement of the anterior fontanelle has also been observed in children with a brachycephalic or acrocephalic (abnormally high) cranium (50, 51).

### 4.4 Limitations

Some limitations in this study should be addressed. One limitation of the study is the relatively small sample size which restricts the generalizability of the findings. Non-brachycephalic control group was heterogenous including various breeds in contrast to brachycephalic group containing only Chihuahuas. Thus, in addition to cranial morphology, breed-, genetic-, and body-size related factors might have influenced the occurrence and size of the bregmatic fontanelle in the heterogenous non-brachycephalic group. Furthermore, in both groups, some puppies originated from the same litter and hence shared genetic, maternal, gestational, and environmental factors. This might have caused pseudoreplication and underestimated standard errors and is a limitation of the study especially in the non-brachycephalic control group. Exclusions of puppies from different analyses might have caused selection bias. Exclusion from micro-CT or MRI was due to limited availability of the imaging modalities. Micro-CT was missing from three Chihuahuas which might have underestimated the number of cranial fontanelles observed in Chihuahua group. Inability to obtain histological samples might have over- or underestimate the number of cranial fontanelles detected in the groups, however, this involved both groups. Molding, i.e., overriding, of cranial sutures to allow the cranium to accommodate passage through the birth canal can occur in newborn puppies. Molding might have been more severe in puppies that have been born by natural parturition or in puppies that have been stuck in the birth canal before Cesarean section. In our study population, molding might have masked small fontanelles, had a minor effect on craniometric measurements and underestimated the width of the bregmatic fontanelle. There was no significant difference in the modes of delivery between the groups, hence molding most likely affected both groups equally. Lack of MRI might have limited the evaluation of the brain in some puppies. Although CT images were available for all puppies, MRI images might have been more accurate in evaluating the brain tissue (52). Furthermore, histological samples of the brain could not be obtained due to damage of the brain caused by sampling of fontanelle sites, limiting evaluation of the brain. Space-occupying lesions can affect cranial ossification and cause resorption of cranial bone (53). If such lesions were present, they might have caused abnormal widening of the bregmatic fontanelle. To reduce this risk, CT images were examined in all puppies for large fluid-filled and hemorrhagic lesions which can be detected in CT images (54, 55). Finally, histological samples used to confirm the presence of cranial fontanelles might have failed to detect small fontanelles. If the histological sample had missed the lesion, it would have led to a false-negative result. This could have underestimated the occurrence of very small cranial fontanelles.

## 5 Conclusion

Based on our results, bregmatic fontanelle occurs more frequently and is relatively wider in newborn Chihuahuas than in non-brachycephalic puppies. Cranial fontanelles located outside the bregma were not detected in non-brachycephalic puppies and were rarely detected in Chihuahuas which differs from the findings previously reported in adult Chihuahuas. This finding indicates that bregmatic fontanelle is a persistent fontanelle. However, our findings indirectly suggest that at least some of the persistent fontanelles located outside the bregma observed in adult Chihuahuas might develop during cranial growth possibly due to bone resorption. Furthermore, our results indicate that the cranial shape of newborn Chihuahuas differs from that of non-brachycephalic puppies, with Chihuahuas exhibiting brachycephalic cranial shape already at birth. A higher degree of brachycephaly (i.e., shorter and higher cranium) was associated with a relatively wider bregmatic fontanelle, suggesting that cranial shape might be associated with cranial ossification already during the fetal period.

## Data Availability

The raw data supporting the conclusions of this article will be made available by the authors, without undue reservation.
